# Brain Metastases Status and Immunotherapy Efficacy in Advanced Lung Cancer: A Systematic Review and Meta-Analysis

**DOI:** 10.3389/fimmu.2021.669398

**Published:** 2021-07-14

**Authors:** Hao Hu, Zhi-Yong Xu, Qian Zhu, Xi Liu, Si-Cong Jiang, Ji-Hua Zheng

**Affiliations:** ^1^ Department of Radiation Therapy, General Hospital of Southern Theater Command, Guangzhou, China; ^2^ The Second Clinical Medical School, Guangzhou University of Chinese Medicine, Guangzhou, China; ^3^ Department of Intensive Care Unit, Sun Yat-sen University Cancer Center, Guangzhou, China; ^4^ Department of Thoracic Surgery, Jiangxi Cancer Hospital of Nanchang University, Nanchang, China

**Keywords:** brain metastases, efficacy, immunotherapy, lung cancer, programmed cell death ligand 1

## Abstract

**Background:**

Brain metastases (BMs) indicate poor outcomes and are commonly excluded in immunotherapy clinical trials in advanced lung cancer; moreover, the effect of BM status on immunotherapy efficacy is inconsistent and inconclusive. Therefore, we conducted a meta-analysis to assess the influence of BM status on immunotherapy efficacy in advanced lung cancer.

**Methods:**

Electronic databases and all major conference proceedings were searched without language restrictions according to the Preferred Reporting Items for Systematic Reviews and Meta-analyses guidelines. We extracted randomized clinical trials on lung cancer immunotherapy that had available overall survival (OS) and/or progression-free survival (PFS) data based on the BM status. All analyses were performed using random effects models.

**Results:**

Fourteen randomized clinical trials with 9,089 patients were identified. Immunotherapy conferred a survival advantage to BM patients [OS-hazard ratio (HR), 0.72; 95% confidence interval (CI), 0.58–0.90; P = 0.004; and PFS-HR, 0.68; 95% CI, 0.52–0.87, P = 0.003]. Non-BM patients could also derive a survival benefit from immunotherapy (OS-HR, 0.76; 95% CI, 0.71–0.80; P <0.001; and PFS-HR, 0.68; 95% CI, 0.56–0.82, P <0.001). The pooled ratios of OS-HRs and PFS-HRs reported in BM patients *versus* non-BM patients were 0.96 (95% CI, 0.78–1.18; P = 0.72) and 0.97 (95% CI, 0.79–1.20; P = 0.78), respectively, indicating no statistically significant difference between them. Subsequent sensitivity analyses did not alter the results. Subgroup analyses according to tumor type, line of therapy, immunotherapy type, study design, and representation of BM patients reconfirmed these findings.

**Conclusion:**

We demonstrated that BM status did not significantly influence the immunotherapy efficacy in lung cancer, suggesting that both BM and non-BM patients could obtain comparable benefits.

**Systematic Review Registration:**

https://www.crd.york.ac.uk/prospero/, identifier (CRD42020207446).

## Introduction

Brain metastases (BMs) are common (approximately 20–40% of cases) and potentially devastating complications in advanced lung cancer, leading to a decreased quality of life and extremely poor prognosis (1, 2). Moreover, the survival benefit of conventional treatment options (e.g. radiotherapy, surgery, and systemic therapy) for BMs patients is limited (3). Thus, new effective therapies to improve the outcomes of BMs patients in lung cancer are warranted.

Recently, immunotherapy has revolutionized lung cancer treatment, resulting in global regulatory approvals and widespread use of such agents in the current clinical practice (4–8). Although many literatures focus on immunotherapy in lung cancer, whether the efficacy of lung cancer immunotherapy differs based on the BM status remains unclear, mainly because of limited data in this area, particularly on the BM patients. First, the low enrollment rate of BM patients makes it unfeasible to recruit sufficient participants to observe the differences. Second, BMs may negatively affect outcomes in patients treated with immunotherapy, and these patients are typically excluded in clinical trials, partly due to poor drug transport across the blood–brain barrier, the risk of brain pseudo-progression, and the use of high-dose corticosteroids (9–11). Third, few studies have conducted a subgroup analysis based on BM status even if the BM patients are included in the immunotherapy trials. Given the poor prognosis of BM patients and potential negative effect on outcomes, there is a clear need to evaluate whether immunotherapy has comparable efficacy between BM and non-BM patients.

Previous randomized controlled trials (RCTs) have presented conflicting findings in the BM patients with lung cancer (4–6). A prior meta-analysis (12) evaluated the clinical efficacy of lung cancer immunotherapy in the BM patients; however, whether the benefits of these agents vary between BM and non-BM patients has not been adequately assessed, largely because of the scarce trials published, as well as the small sample sizes analyzed. Moreover, an analysis of disproportionately fewer BM patients (6.2–17.5%) in trials may result in unreliable or even false results (13, 14). Nevertheless, the statistical power of meta-analyses of such trials may be enhanced by integrating these small subgroup analyses, hence drawing more accurate results.

Now that the results of several RCTs on immunotherapy according to BM status have become increasingly available, we therefore conducted a meta-analysis to examine the effect of BM status on immunotherapy efficacy in advanced lung cancer.

## Methods

### Search Strategy

We made a predetermined protocol (PROSPERO registration number: CRD42020207446) to perform a systematic literature search and meta-analysis according to the Preferred Reporting Items for Systematic Reviews and Meta-Analyses guidelines (15). The PubMed, Cochrane Library, and EMBASE databases were searched for phase 2 and 3 RCTs on lung cancer immunotherapy [i.e., anti-programmed cell death 1 or programmed cell death ligand 1 (PD-1/PD-L1) inhibitors] from the inception to June 1, 2020 without language restrictions. The abstracts and presentations from the American Society of Clinical Oncology, World Conference on Lung Cancer, European Society for Medical Oncology, and American Association for Cancer Research were also reviewed from January 1, 2015 to December 1, 2020. Moreover, the references of the identified articles were reviewed (further information is listed in **Supplementary Table 1**).

### Study Selection

The inclusion criteria were: 1) phase 2 and 3 RCTs investigating new immunotherapy agents against a control regimen (conventional standard therapy) in patients with advanced lung cancer; and 2) available data on hazard ratios (HRs) for overall survival (OS) and/or progression-free survival (PFS) based on BM status (with or without BMs). Conversely, the exclusion criteria were: 1) studies that explored only BM or non-BM patients; 2) single-arm and non-randomized studies (i.e., retrospective or prospective observational cohort); 3) studies without OS and PFS outcomes data according to BM status; and 4) an immunotherapy agent in both arms. We included the most recent and/or most complete trial if duplicate clinical trials were identified.

### Data Extraction and Risk of Bias Assessment

For each study, the study name, phase, stage, blinding, histological type, number of patients, BM distribution, treatment characteristics (line of therapy, study drugs, median follow-up time), and survival outcomes data (OS and PFS) according to the BM status were extracted. We adopted the Cochrane Collaboration tool to estimate the risk of bias (16), and applied the 5-point Jadad score to evaluate the methodological quality of the studies (an overall score of 0 indicated the worst methodological quality, and 5 indicated optimal methodological quality) (17). Funnel plots, Egger’s test, and Begg’s test were conducted to test the risk of publication bias.

### Statistical Analyses

We used the same method of determining the difference in immunotherapy efficacy between BM and non-BM patients to avoid the risk of ecological bias, as previously reported (18, 19). First, we calculated an interaction trial-specific HR for each study (the ratio of HR in BM patients to HR in non-BM patients). Then, we used a random effects model to combine the trial-specific HR ratios across trials. Study heterogeneity was investigated with the Q test, which was quantified using the I^2^ test (20). All analyses were performed using random effects models and Stata version 14.0 (StataCorp, College Station, TX). Statistically significant was set at *P* values <0.05 in the two-tailed tests.

### Subgroup and Sensitivity Analyses

The pre-specified subgroup analyses included tumor type [non-small cell lung cancer (NSCLC) *vs*. small cell lung cancer (SCLC)], study design [immunotherapy *vs*. standard of care (SOC) alone, immunotherapy + SOC *vs*. SOC alone], line of therapy (first-line *vs*. second- or later-line), immunotherapy type (anti-PD-1 *vs*. anti-PD-L1), and the proportion of BM patients in each study (<10% *vs*. ≥10 of the study cohort). We tested the subgroups using the χ² test and excluded subgroups that included less than two studies to avoid possible selection bias. Sensitivity analysis was conducted by excluding the trials that recruited patients with particular conditions, trials with a unique study design, and trials that used a fixed-effects model.

## Results

### Search Results

**Figure 1:**
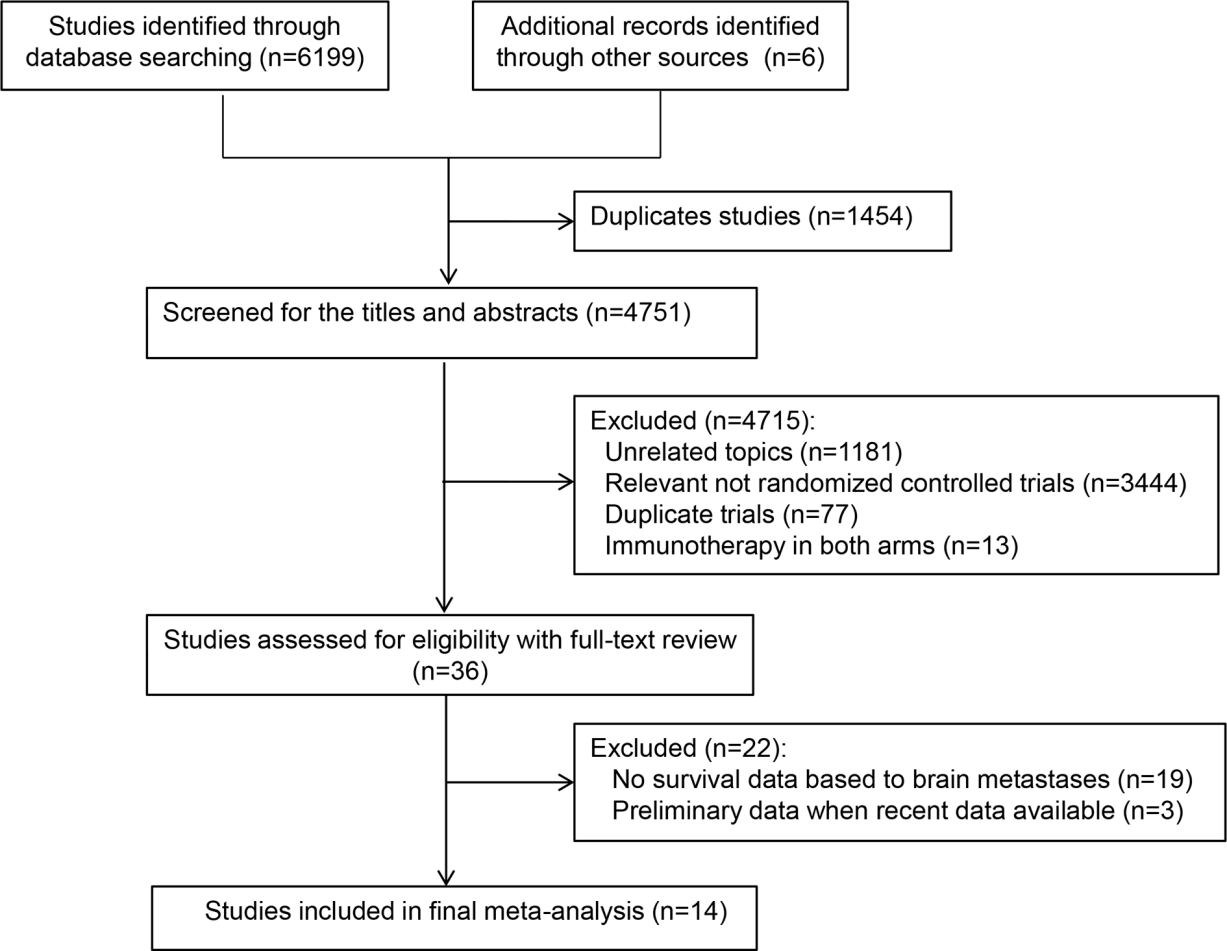
Preferred Reporting Items for Systematic Reviews and Meta-analyses diagram.

Database and manual searches yielded a total of 6,205 references, and 1,454 studies were excluded because of duplications. We then checked the titles and abstracts, and 4,715 studies were excluded because they were not in line with the inclusion criteria. After screening the full-text of the remaining 36 potentially eligible studies, we identified 14 relevant clinical trials for the final analysis (4, 6–8, 21–30). Of these, one trial (26) reported two treatment arms with different regimens (durvalumab plus platinum–etoposide with or without tremelimumab). Finally, a total of 15 independent cohorts from the 14 included trials were recorded (**Table 1**). **Figure 1** presents the study selection flowchart.

**Table 1:** Main Characteristics of the Included 14 Trials.

Study name (phase, dominant ethnicity)	Tumor type	Line of therapy	Intervention (No.)	Control treatment (No.)	Age, median (Range or IQR), y	Follow-up, median (Range or IQR), mo	Randomization stratified by BMs status	BMs status (No.)		Jadad score
								No	Yes	
CheckMate 057 (4), (3, White)	NSCLC	>1	Nivolumab (n = 292)	Docetaxel (n = 290)	62 (21–85)	13.2 (NR)	No	514	68	3
KEYNOTE-024 (23), (3, White)	NSCLC	1	Pembrolizumab (n = 154)	CTx (n = 151)	65 (33–90)	25.2 (20.4–33.7)	No	277	28	3
JAVELIN Lung 200 (21), (3, White)	NSCLC	>1	Avelumab (n = 396)	Docetaxel (n = 396)	63 (57–69)	18.3 (12.9–22.9)	No	713	79	3
OAK (6), (3, White)	NSCLC	>1	Atezolizumab (n = 613)	Docetaxel (n = 612)	64 (25–85)	26 (NR)	No	1,107	118	3
KEYNOTE-189 (7), (3, White)	NSCLC	1	Pembrolizumab+CTx (n = 410)	CTx (n = 206)	64 (34–84)	23.1 (18.6–30.9)	No	508	108	5
CheckMate 227 (22), (3, White)	NSCLC	1	Nivolumab+ ipilimumab (n = 583)	CTx (n = 583)	64 (26–87)	29.3 (NR)	No	1,051	115	3
CheckMate 078 (24), (3, Asian)	NSCLC	>1	Nivolumab (n = 338)	Docetaxel (n = 166)	60 (27–78)	8.8 (NR)	No	432	72	3
SHR-1210-303 (25), (3, Asian)	NSCLC	1	Camrelizumab+CTx (n = 205)	CTx (n = 207)	60 (24–71)	11.9 (NR)	No	395	17	3
Checkmate 9LA (27), (3, White)	NSCLC	1	Nivolumab+ ipilimumab +CTx (n = 361)	CTx (n = 358)	65 (26–86)	12.7 (NR)	No	597	122	3
IMpower133 (8), (3, White)	SCLC	1	Atezolizumab+ CTx (n = 201)	CTx (n = 202)	64 (26–90)	13.9 (NR)	Yes	368	35	5
CASPIAN (26), (3, White)	SCLC	1	Durvalumab+ CTx (268)	CTx (n = 269)	63 (28–88)	25.1 (0.1–33.7)	No	482	55	3
			Durvalumab+ tremelimumab+ CTx (n = 268)					472	65	
Keynote 604 (28), (3, White)	SCLC	1	Pembrolizumab + CTx (n = 228)	CTx (n = 225)	65 (24–83)	21.6 (16.1–30.6)	No	398	55	5
ORIENT-11 (30), (3, Asian)	NSCLC	1	Sintilimab +CTx (n = 266)	CTx (n = 131)	61 (30–75)	8.9 (NR)	No	339	58	5
EMPOWER-Lung 1 (29), (3, Asian)	NSCLC	1	Cemiplimab (n = 356)	CTx (n = 356)	63 (31–84)	13.1 (NR)	No	627	83	3

Data are presented as n (%), and median (range), unless otherwise stated.

BMs, brain metastases; CTx, chemotherapy; NR, not reported; IQR, interquartile range; NSCLC, non-small-cell lung cancer; SCLC, small-cell lung cancer; HR, hazard ratio; CI, confidence interval.

### Main Characteristics of the Identified Trials

All included trials were randomized multi-center international phase 3 trials; four were double-blind trials (7, 8, 28, 30), and only one trial performed randomization stratified by BM status (8). There were 11 trials (78.6%) with patients with NSCLC (4, 6, 7, 21–25, 27, 29, 30), and three (27.3%) with those with SCLC (8, 26, 28). Most studies (71.4%) evaluated immunotherapy in the first-line setting (7, 8, 22, 23, 25–30), whereas four trials (28.6%) assessed the efficacy in the second- or later-line setting (4, 6, 21, 24). Seven trials (50%) included the immunotherapy-chemotherapy combination *vs*. SOC alone (7, 8, 25–28, 30), both of which recruited patients with advanced or metastatic disease.

The study size ranged from 305 to 1,225 patients. Among all the 9,089 patients included, 1,051 (11.6%) were BM patients, and 8,038 (88.4%) were non-BM patients; notably, the proportion of BM patients differ widely between studies (4.1% to 17.5% of all cancers). The median follow-up duration varied between 8.8 months and 29.3 months, and most trials (78.6%) had a more than 12-months median follow-up (4, 6–8, 21–23, 26–29). Seven studies (50%) evaluated OS as the primary endpoint (4, 6, 21, 22, 24, 26, 27), three (23, 25, 30) assessed OS as the secondary endpoint (the primary endpoint was PFS), and four (7, 8, 28, 29) chose OS and PFS as dual primary endpoints. Moreover, 50% of the included studies allowed patients who presented disease progression in the control group to crossover to the immunotherapy group. The main characteristics of the 14 included trials are listed in **Table 1** and **Supplementary Table 2**.

### Bias Assessment

As summarized in **Supplementary Table 3**, all trials received moderate-to-high quality (Jadad scores of 3–5). Minimal or no publication bias for OS and PFS were detected *via* the funnel plots, respectively (**Supplementary Figure 1**). Moreover, additional tests failed to find any publication bias for the outcome OS (Egger’s test P = 0.34; Begg’s test P = 0.43) or PFS (Egger’s test P = 0.65; Begg’s test P = 1).

### The Relationship Between BM Status and OS Outcomes

**Figure 2:**
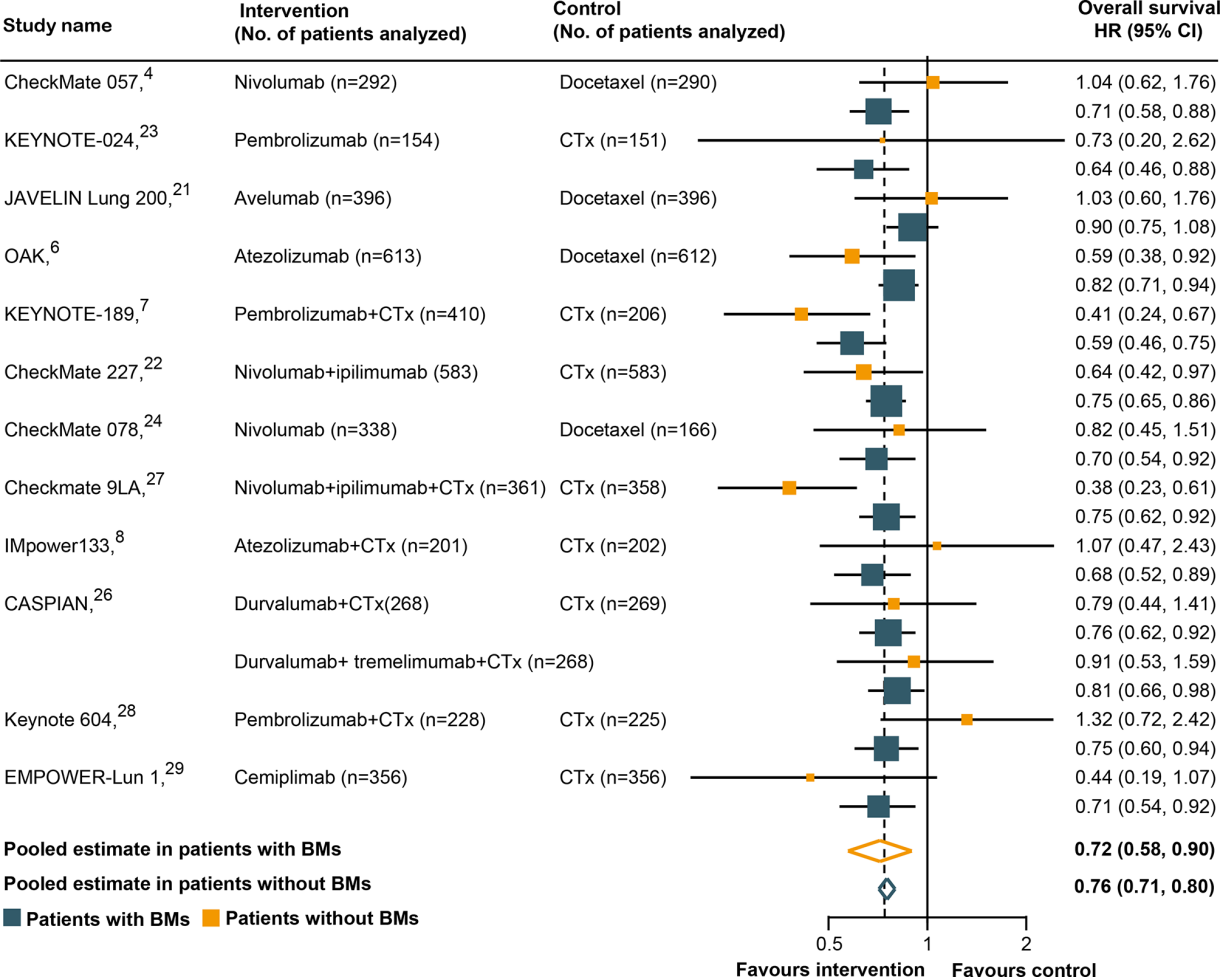
Hazard ratios for overall survival when comparing immunotherapy to control treatment.

All trials except two (25, 30) had available OS data according to the BM status and were included in the pooled estimates for such an endpoint. As shown in **Figure 2**, immunotherapy could reduce the risk of death for BM patients, as compared with SOC systemic therapies (HR, 0.72; 95% CI, 0.58–0.90; P = 0.004). A similar result was uncovered for non-BM patients (HR, 0.76; 95% CI, 0.71–0.80; P <0.001). However, a statistically significant heterogeneity was found in the BM patients (χ^2^ = 22.79; P = 0.03; I^2^ = 47.3%), but not in the non-BM patients (χ^2^ = 11.74; P = 0.467; I^2^ = 0%). The pooled HR for OS in all patients, including both BM and non-BM patients, was 0.74 (95% CI, 0.69–0.79; P <0.001). However, we failed to discover any statistically significant differences in OS between BM and non-BM patients **(**P = 0.72 for interaction) (**Table 2**). The pooled ratio of OS-HRs in BM *versus* non-BM patients reported in each trial was 0.96 (95% CI, 0.78–1.18) (**Supplementary Figure 2**). Moreover, the sensitivity analysis using a fixed-effects model showed that the results did not change. KEYNOTE-024 (23) recruited only patients with PD-L1 ≥50%, whereas CheckMate 227 (22), CASPIAN (26), and Checkmate 9LA (27) have unique study designs, which included an anti-cytotoxic T-lymphocyte antigen 4 agents (tremelimumab or ipilimumab). The sensitivity analysis was performed to separately exclude KEYNOTE-024 (23), CheckMate 227 (22), CASPIAN (26), and Checkmate 9LA (27); nevertheless, the results remained unchanged (**Supplementary Table 4**). The results of the subgroup analyses for OS outcomes are summarized in **Table 2**. No statistically significant differences in the OS outcome were demonstrated between BM and non-BM patients based on tumor type, line of therapy, immunotherapy type, and study design. Finally, we further evaluated the effect of the prevalence of BMs in the study cohort and found no statistically significant differences between these subgroups.

**Table 2:** Analyses of Pooled Hazard Ratios for OS Outcomes by Subgroup.

Variables	Study, No. (%)	Participants, No.		Pooled HR (95% CI) for immunotherapy *vs* SOC		P value for interaction
		BMs	non-BMs	BMs	non-BMs	
Overall	13	976	7,304	0.72 (0.58–0.90)	0.76 (0.71–0.80)	0.72
Tumor type						
NSCLC	9 (69)	793	5,826	0.63 (0.49–0.82)	0.75 (0.69–0.81)	0.19
SCLC	4 (31)	183	1,478	0.99 (0.72–1.34)	0.76 (0.68–0.85)	0.13
Study design						
immunotherapy *vs* SOC	7 (54)	563	4,721	0.74 (0.60–0.91)	0.77 (0.71–0.83)	0.73
immunotherapy + SOC *vs* SOC	6 (46)	413	2,583	0.71 (0.46–1.09)	0.73 (0.67–0.80)	0.91
Line of therapy						
first-line	9 (69)	639	4,538	0.67 (0.49–0.90)	0.73 (0.68–0.78)	0.54
second- or later-line	4 (31)	337	2,766	0.83 (0.62–1.10)	0.80 (0.72–0.89)	0.75
Immunotherapy type						
anti-PD-1	8 (62)	651	4,404	0.66 (0.47–0.92)	0.71 (0.66–0.77)	0.66
anti-PD-L1	5 (38)	325	2,900	0.81 (0.63–1.03)	0.81 (0.74–0.88)	0.97
BMs proportion						
<10	5 (38)	375	3,516	0.73 (0.57–0.93)	0.78 (0.71–0.86)	0.52
≥10	8 (62)	601	3,788	0.70 (0.50–0.98)	0.73 (0.67–0.79)	0.87

NSCLC, non–small cell lung cancer; SCLC, small cell lung cancer; PD-1, programmed cell death 1; PD-L1, programmed cell death ligand 1; SOC, standard of care chemotherapy; BMs, brain metastases; OS, overall survival; HR, hazard ratio; CI, confidence interval.

### The Relationship Between BM Status and PFS Outcomes

**Figure 3:**
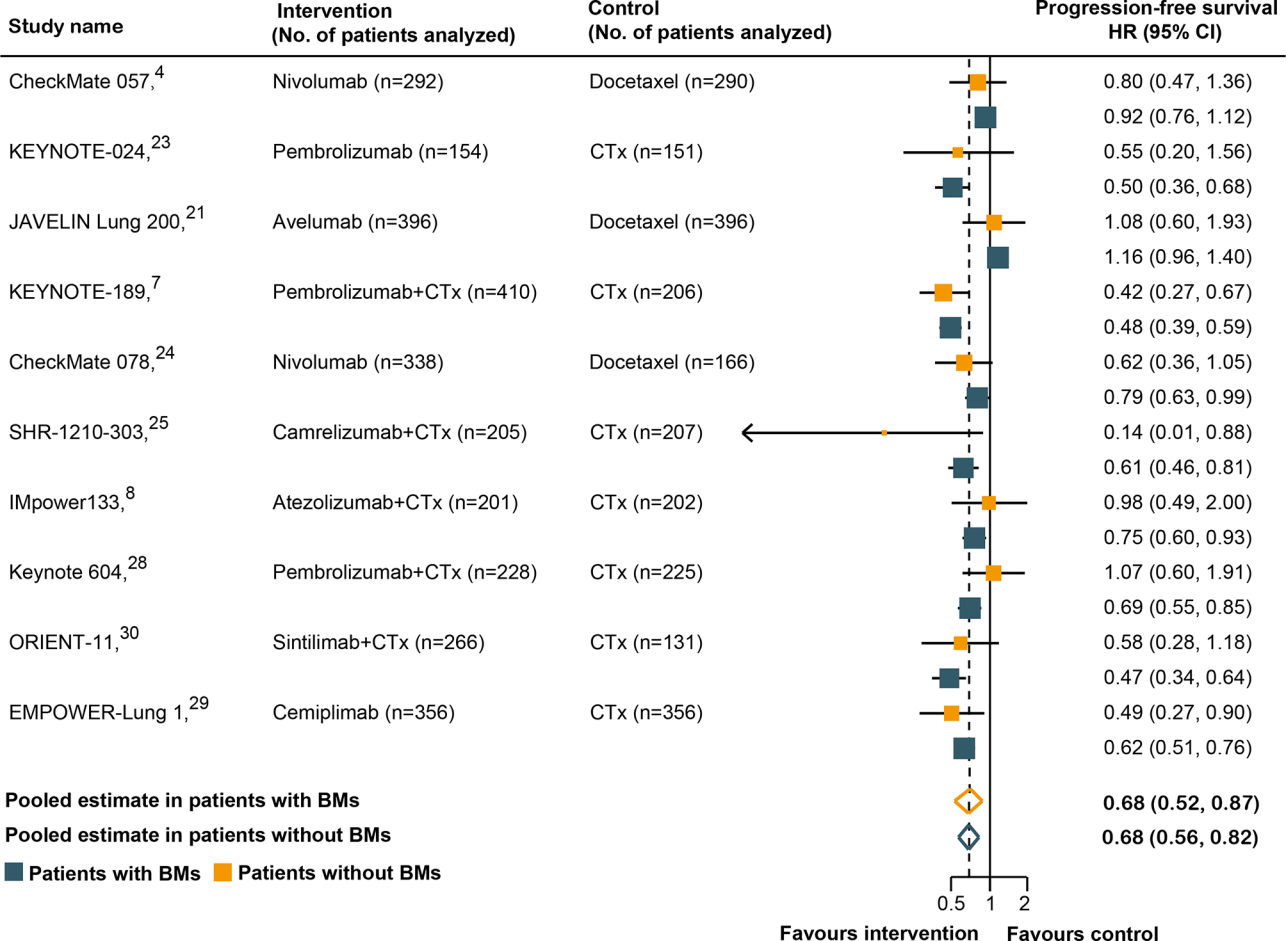
Hazard ratios for progression-free survival when comparing immunotherapy to control treatment.

Ten of the 14 RCTs were included in the pooled estimation because they had available PFS data according to the BM status (4, 7, 8, 21, 23–25, 28–30). As shown in **Figure 3**, BM patients treated with immunotherapy experienced a significantly lower risk of progression as compared with those treated with SOC systemic therapies (HR, 0.68; 95% CI, 0.52–0.87; P = 0.003). In non-BM patients, the PFS benefit obtained with immunotherapy compared to that with SOC systemic therapies was similar (HR, 0.68; 95% CI, 0.56–0.82; P <0.001). However, a substantial heterogeneity was detected in non-BM patients (χ^2^ = 76.17; P <0.001; I^2^ = 85.4%), but not in BM patients (χ^2^ = 13.98; P = 0.12; I^2^ = 35.6%). Overall, the pooled HR for PFS in all patients, including both BM and non-BM patients, was 0.68 (95% CI, 0.56–0.82; P <0.001). However, no PFS benefit difference between BM and non-BM patients was found (P = 0.78 for interaction) (**Table 2**). The pooled ratio of PFS-HRs in BM *versus* non-BM patients reported in each trial was 0.97 (95% CI, 0.79–1.20) (**Supplementary Figure 3**). As shown in **Supplementary Table 4**, sensitivity analysis with a fixed-effects model demonstrated that the results were not altered. The results of the sensitivity analysis were also consistent after omitting the trial by KEYNOTE-024 (23). **Table 3** shows the results of the subgroup analyses for the association between BM status and PFS outcomes. For subgroups including tumor type, line of therapy, immunotherapy type, and study design, the PFS benefit obtained from immunotherapy *vs*. SOC did not differ between BM- and non-BM patients. In addition, the prevalence of BM in the study cohort did not show significant differences within the subgroups (**Table 3**).

**Table 3:** Analyses of Pooled Hazard Ratios for PFS Outcomes by Subgroup.

Variables	Study, No. (%)	Patients, No.		Pooled HR (95% CI) for immunotherapy *vs* SOC		P value for interaction
		BMs	non-BMs	BMs	non-BMs	
Overall	10	603	4,571	0.68 (0.52–0.87)	0.68 (0.56–0.82)	0.78
Tumor type						
NSCLC	8 (80)	513	3,805	0.61 (0.46–0.79)	0.67 (0.52–0.85)	0.25
SCLC	2 (20)	90	766	1.03 (0.66–1.61)	0.72 (0.62–0.84)	0.13
Study design						
immunotherapy *vs* SOC	5 (50)	330	2,563	0.71 (0.54–0.93)	0.78 (0.59–1.02)	0.25
immunotherapy + SOC *vs* SOC	5 (50)	273	2,008	0.65 (0.40–1.06)	0.60 (0.49–0.72)	0.47
Line of therapy						
first-line	7 (70)	384	2,912	0.61 (0.43–0.87)	0.59 (0.51–0.68)	0.74
second- or later-line	3 (30)	219	1,659	0.80 (0.58–1.10)	0.95 (0.77–1.18)	0.37
Immunotherapy type						
anti-PD-1	8 (80)	489	3,490	0.61 (0.47–0.79)	0.63 (0.53–0.75)	0.61
anti-PD-L1	2 (20)	114	1,081	1.04 (0.66–1.63)	0.94 (0.61–1.44)	0.77
BMs proportion						
<10	4 (40)	159	1,753	0.83 (0.51–1.37)	0.73 (0.50–1.05)	0.97
≥10	6 (60)	444	2,818	0.62 (0.47–0.83)	0.65 (0.52–0.80)	0.70

NSCLC, non–small cell lung cancer; SCLC, small cell lung cancer; PD-1, programmed cell death 1; PD-L1, programmed cell death ligand 1; SOC, standard of care chemotherapy; BMs, brain metastases; PFS, progression-free survival; HR, hazard ratio; CI, confidence interval.

## Discussion

To the best of our knowledge, the present study is the first meta-analysis to compare the long-term outcomes of immunotherapy between BM and non-BM patients with advanced lung cancer. The results demonstrated no difference in OS and PFS between BM and non-BM patients. Moreover, subsequent sensitivity analyses did not alter the results. Furthermore, subgroup analyses according to tumor type, line of therapy, immunotherapy type, and study design also demonstrated no significant BM-associated differences in the efficacy. Hence, our study suggests that immunotherapy is preferable to conventional SOC therapy for treating both BM and non-BM patients with advanced lung cancer. Moreover, the BM status did not significantly affect the efficacy of PD-L1-based immunotherapy, indicating that both BM and non-BM patients could obtain comparable survival benefits from lung cancer immunotherapy. Therefore, the BM status should not be the only decisive factor for the use of PD-L1-based immunotherapy treatment during routine clinical practice and in future research.

Despite lung cancer immunotherapy has received extensive attention, the efficacy of these agents in BM patients was still uncertain, mainly because of limited information available in published trials. A previously published meta-analysis (12) included only three trials with 259 BM patients with NSCLC, and concluded that BM patients could obtain OS benefit from immunotherapy in combination with chemotherapy rather than immunotherapy alone. Nevertheless, their study may have been biased by the small sample sizes, with limited trials analyzed. Our study, which included 13 cohorts with 976 BM patients, is the largest study to test the immunotherapy efficacy of lung cancer among BM patients. We found that the risk of death in BM patients was significantly reduced by 26% when treated with immunotherapy monotherapy (**Table 2**); however, the OS benefit of immunotherapy in combination with chemotherapy was marginal (HR, 0.71; 95% CI, 0.46–1.09), partially due to the relatively small sample size, and the lack of statistical power to discover a significant difference. However, there are also few studies inconsistent with our results. A recent multicenter retrospective study enrolled patients with several types of metastatic cancer, including NSCLC who received immunotherapy (31). The study demonstrated that patients with BM had worse PFS and OS than did those without BMs. Similarly, a prior systematic review and meta-analysis inferred that BM were independent predictors of the poor survival outcomes in advanced NSCLC patients treated with PD-1-based immunotherapy (32). However, their conclusions might be limited by the heterogeneity of treatment characteristics between different studies and the inherent biases owing to the retrospective nature of most of the included studies in these reviews. BM patients are known to have an unfavorable prognosis in lung cancer, with a 1-year survival rate of <10% (33). They tend to have a range of symptoms (e.g., altered mental status, visual impairments with headaches, and fatigue), which can lead to psychological, social, and physical debilitation, as well as greater social and economic burdens (34). Therefore, this challenge emphasizes the further clinical implication and importance of the current research. BMs are commonly considered to be a predictor of poor outcomes in patients with advanced lung cancer treated with PD-L1-based immunotherapy (32). In this study, we demonstrated that BMs did not negatively influence the efficacy of lung cancer immunotherapy. Our findings may be explained in several ways. First, the normal brain has been long recognized as an ‘‘immune privileged’’ organ in the body because the blood–brain barrier could prevent it from immune cell entry (35). However, the blood–brain barrier is damaged or influenced in BM patients and can allow substantial immune cells (e.g., peripherally activated T cells) to enter and/or infiltrate (36). In addition, the change in the blood–brain barrier makes it possible for immunotherapy agents to function in the brain. In support of this, specimens of BMs exhibit dense infiltrates of tumor-infiltrating lymphocytes and is correlated with favorable survival outcomes, further providing the basis for treating BM patients with these agents (37, 38). Second, resected BMs have a higher tumor mutation burden (TMB) than paired primary lung tumors (39, 40). Prior studies (41, 42) have suggested that TMB is a promising predictive biomarker for immunotherapy in diverse cancers, including lung cancer. Hence, high tumor mutation load in BMs and increased frequency of neoantigens may contribute to an improved response to lung cancer immunotherapy (39). Third, higher PD-L1 expression in tumor cells has been noted in lung cancer BM than in matched primary tumors (43). As previously demonstrated (19), the survival benefit from immunotherapy is PD-L1-dependent, and patients with high-level PD-L1 expression had a greater survival advantage. Accordingly, our results may be partly attributable to the overexpression of PD-L1 in BM patients. Fourth, patients with active or untreated BMs and patients who require systemic steroids (poorer prognostic factors) are usually excluded from immunotherapy trials (10, 44). The observed survival benefits in BM patients cannot be ruled out because of the more favorable prognostic profile in these patients. Consistently, several previous retrospective studies demonstrated that BMs did not significantly correlate with survival outcomes in advanced NSCLC patients treated with nivolumab, an anti-PD1 agent (45–47).

Previous studies (48, 49) have also demonstrated that anti-PD-1 agents show better anti-cancer effect than anti-PD-L1 agents in the treatment of advanced cancer, including lung cancer, partly owing to the inherent discrepancy among them. In the present study, we found a consistently better efficacy in BM patients treated with anti-PD-1 agents: anti-PD-1 agents significantly improved OS and PFS outcomes compared with conventional SOC therapy in BM patients, whereas anti-PD-L1 agents did not (**Tables 2** and **3**). Our results suggest a possible superior anti-tumor effect of anti-PD-1 agents in the treatment of BM patients, although this finding remains unclear from the sample size in this analysis. Therefore, large RCTs are essential for investigating the relative survival advantage of different immunotherapy agents in BM patients to identify best treatment. Notably, in SCLC, immunotherapy was not effective in improving OS and PFS in BM patients. Nevertheless, the available data were only from a small number of BM patients, and the observed wide CIs for the calculated HRs in these patients prevented us from drawing definitive conclusions.

This study has some limitations. Of note, our findings are based on published trials, rather than individual patient data. Furthermore, patients included in our study had treatable, stable, and asymptomatic BMs, rather than having untreatable, active, or symptomatic BMs. However, a recent phase II trial (50) has revealed that pembrolizumab, an anti-PD-1 inhibitor, showed consistent brain and extra-cerebral responses in patients with NSCLC, indicating that immunotherapy can be active in patients with active BMs. Other trials (51–53) have also found that immunotherapy agents are active in patients with active melanoma BMs. Additionally, we cannot rule out that some factors other than BMs are distributed differently between BM and non-BM patients, and that these factors might affect our results. Finally, previous reports have investigated the prognosis of BM patients in several types of metastatic cancer, and the prognostic factors varied between different tumor types. For instance, a study established a nomogram based on 3,522 patients from the Surveillance, Epidemiology, and End Results database, and demonstrated that age, marital status, T stage, N stage, race, and gender were independent predictors of survival in SCLC patients with BM (54). Meanwhile, a cohort of 227 patients with BM from colorectal cancer proposed that age, performance status, BM site, and BM number were independent prognostic factors for survival (55). However, evidences from NSCLC patients with BM suggested that BM number did not influence the survival outcome (56, 57). In our study unfortunately, the included trials were not conducted specifically to evaluate the intracranial efficacy of lung cancer immunotherapy, and thus several detail data related to BM had not been reported in published clinical trials. Therefore, we could not assess the effect of immunotherapy on the reduced size and severity of BM. Future studies on BM patients are needed to evaluate the intracranial efficacy of immunotherapy in advanced lung cancer.

In the current meta-analysis of all available randomized trials of lung cancer immunotherapy, we demonstrated that BM and non-BM patients could derive similar survival advantages. We recommend that BM status may not be the only consideration when deciding whether to offer immunotherapy to patients with advanced lung cancer in routine clinical practice and future clinical trial designs.

## Data Availability Statement

The original contributions presented in the study are included in the article/**Supplementary Material**. Further inquiries can be directed to the corresponding author.

## Author Contributions

J-HZ had full access to all of the data in this study and accepts responsibility for the integrity of the data and the accuracy of the data analysis. Concept and design: HH, ZY-X, and QZ. Acquisition, analysis, or interpretation of data: All authors. Drafting of the manuscript: All authors. Critical revision of the manuscript for important intellectual content: All authors. Statistical analysis: All authors. Administrative, technical, or material support: All authors. Supervision: HH, J-HZ. All authors contributed to the article and approved the submitted version.

## Funding

This study was supported by the Science and Technology Planning Project of Guangzhou, China (No. 202102080513), and the Medical Scientific Research Foundation of Guangdong Province, China (No. A2021279).

## Conflict of Interest

The authors declare that the research was conducted in the absence of any commercial or financial relationships that could be construed as a potential conflict of interest.
