# Supplementary material for: Brain Metastases Status and Immunotherapy Efficacy in Advanced Lung Cancer: A Systematic Review and Meta-Analysis
Source: Front Immunol. 2021 Jul 14;12:669398. doi: 10.3389/fimmu.2021.669398 (PMC8316922; doi:10.3389/fimmu.2021.669398)
Supplement: Supplementary file 1 [file DataSheet_1.docx]

Supplementary Material

# Supplementary Tables and Figures

## Supplementary Tables

**Supplementary Table 1.** Search strategy for each database

**PubMed: 2,157 results**

#1 "pembrolizumab" [Supplementary Concept] OR "pembrolizumab" [All Fields] OR "lambrolizumab" [All Fields] OR "Keytruda" [All Fields] OR "MK-3475" [All Fields]

#2 "Nivolumab"[Mesh] OR "Nivolumab"[All Fields] OR "Opdivo"[All Fields] OR "ONO-4538"[All Fields] OR "ONO 4538"[All Fields] OR "MDX-1106"[All Fields] OR "MDX 1106"[All Fields] OR "MDX1106"[All Fields] OR "BMS-936558"[All Fields] OR "BMS 936558"[All Fields] OR "SCH-900475"[All Fields] OR "Nivo"[All Fields]

#3 "atezolizumab"[Supplementary Concept] OR "atezolizumab"[All Fields] OR "anti-PDL1"[All Fields] OR "anti-PD1"[All Fields] OR "Tecentriq"[All Fields] OR "RG7446"[All Fields] OR "RG-7446"[All Fields] OR "MPDL3280A"[All Fields]

#4 "avelumab" [Supplementary Concept] OR "avelumab" [All Fields] OR "MSB0010718C" [All Fields]

#5 "durvalumab" [Supplementary Concept] OR "durvalumab" [All Fields] OR "MEDI-4736" [All Fields] OR "MEDI4736" [All Fields]

#6 "Programmed Cell Death 1 Receptor"[Mesh] OR "PD-1"[All Fields] OR "PD-L1"[All Fields] OR "B7-H1 Antigen"[Mesh] OR "Programmed death ligand 1"[All Fields]

#7 #1 OR #2 OR #3 OR #4 OR #5 OR #6

#8 "Lung Neoplasms"[Mesh] OR "lung cancer*" [Title/Abstract] OR "lung carcinoma*" [Title/Abstract] OR "lung malignan*"[Title/Abstract] OR "lung neoplasm*"[Title/Abstract] OR " lung tumo*"[Title/Abstract] OR "pulmonary cancer*"[Title/Abstract] OR "pulmonary carcinom*"[Title/Abstract] OR "pulmonary malignan*"[Title/Abstract] OR "pulmonary neoplasm*"[Title/Abstract] OR "pulmonary tumo*"[Title/Abstract] OR "carcinoma, non-small-cell lung"[MeSH] OR "nonsmall cell lung cancer*"[Title/Abstract] OR "non small cell lung cancer*"[Title/Abstract] OR "non small cell lung carcinoma*"[Title/Abstract] OR "NSCLC"[Title/Abstract] OR "carcinoma, small cell" [MeSH] OR "oat cell carcinoma*"[Title/Abstract] OR "oat cell lung carcinoma*"[Title/Abstract] OR "oat cell lung cancer*"[Title/Abstract] OR "oat cell cancer*"[Title/Abstract] OR "SCLC" [Title/Abstract] OR "small cell lung cancer*"[Title/Abstract] OR "small cell lung carcinom*"[Title/Abstract]

#9 randomized controlled trial [pt] OR controlled clinical trial [pt] OR randomized [tiab] OR placebo [tiab] OR drug therapy [sh] OR randomly [tiab] OR trial [tiab] OR groups [tiab]

#10 animals [mh] NOT humans [mh]

#11 #9 NOT #10

#12 #7 AND #8 AND #11

**EMBASE: 2,560 results**

#1 'pembrolizumab'/exp OR 'pembrolizumab' OR 'lambrolizumab' OR 'keytruda' OR 'mk-3475' OR 'nivolumab'/exp OR 'nivolumab' OR 'opdivo' OR 'ono-4538' OR 'ono 4538' OR 'mdx-1106' OR 'mdx 1106' OR 'mdx1106' OR 'bms-936558' OR 'bms 936558' OR 'sch-900475' OR 'nivo' OR 'atezolizumab'/exp OR 'atezolizumab' OR 'anti-pdl1' OR 'tecentriq' OR 'rg7446' OR 'rg-7446' OR 'mpdl3280a' OR 'avelumab'/exp OR 'avelumab' OR 'msb0010718c' OR 'durvalumab'/exp OR 'durvalumab' OR 'medi-4736' OR 'medi4736' OR 'programmed cell death 1 receptor'/exp OR 'programmed cell death 1 receptor' OR 'pd-1' OR 'pd-l1' OR 'programmed death ligand 1' OR 'b7-h1 antigen'/exp

#2 'lung tumor'/exp OR 'lung cancer*':ab,ti OR 'lung carcinoma*':ab,ti OR 'lung malignan*':ab,ti OR 'lung neoplasm*':ab,ti OR 'lung tumo*':ab,ti OR 'pulmonary cancer*':ab,ti OR 'pulmonary carcinom*':ab,ti OR 'pulmonary malignan*':ab,ti OR 'pulmonary neoplasm*':ab,ti OR 'pulmonary tumo*':ab,ti OR 'lung non small cell cancer'/exp OR 'nonsmall cell lung cancer*':ab,ti OR 'non small cell lung cancer*':ab,ti OR 'nonsmall cell lung carcinoma*':ab,ti OR 'non small cell lung carcinoma*':ab,ti OR 'nsclc':ab,ti OR 'small cell lung cancer'/exp OR 'oat cell carcinoma*':ti,ab OR 'oat cell lung carcinoma*':ti,ab OR 'oat cell lung cancer*':ti,ab OR 'oat cell cancer*':ti,ab OR 'sclc':ti,ab OR 'small cell lung cancer*':ti,ab OR 'small cell lung carcinom*':ti,ab

#3 'crossover procedure':de OR 'double-blind procedure':de OR 'randomized controlled trial':de OR 'singleblind procedure':de OR (random* OR factorial* OR crossover* OR cross NEXT/1 over* OR placebo* OR doubl* NEAR/1 blind* OR singl* NEAR/1 blind* OR assign* OR allocat* OR volunteer*):de,ab,ti

#4 #1 AND #2 AND #3

**Cochrane library: 1,482 results**

#1 MeSH descriptor: [Lung Neoplasms] explode all trees

#2 lung cancer*:ti,ab

#3 lung carcinoma*:ti,ab

#4 lung malignan*:ti,ab

#5 lung neoplasm*:ti,ab

#6 lung tumo*:ti,ab

#7 pulmonary cancer*:ti,ab

#8 pulmonary carcinom*:ti,ab

#9 pulmonary malignan*:ti,ab

#10 pulmonary neoplasm*:ti,ab

#11 pulmonary tumo*:ti,ab

#12 MeSH descriptor: [Carcinoma, Non-Small-Cell Lung] explode all trees

#13 nonsmall cell lung cancer*:ti,ab

#14 non small cell lung cancer*:ti,ab

#15 nonsmall cell lung carcinoma*:ti,ab

#16 non small cell lung carcinoma*:ti,ab

#17 NSCLC:ti,ab

#18 MeSH descriptor: [Carcinoma, Small Cell] explode all trees

#19 oat cell carcinoma*:ti,ab

#20 oat cell lung carcinoma*:ti,ab

#21 oat cell lung cancer*:ti,ab

#22 oat cell cancer*:ti,ab

#23 SCLC:ti,ab

#24 small cell lung cancer*:ti,ab

#25 small cell lung carcinom*:ti,ab

#26 #1 or #2 or #3 or #4 or #5 or #6 or #7 or #8 or #9 or #10 or #11 or #12 or #13 or #14 or #15 or #16 or #17 or #18 or #19 or #20 or #21 or #22 or #23 or #24 or #25

#27 MeSH descriptor: [Nivolumab] explode all trees

#28 (pembrolizumab OR lambrolizumab OR Keytruda OR MK-3475):ti,ab

#29 (PD-1 or PD-L1 or programmed cell death 1 receptor or programmed death ligand 1):ti,ab

#30 MeSH descriptor: [B7-H1 Antigen] explode all trees

#31 MeSH descriptor: [Programmed Cell Death 1 Receptor] explode all trees

#32 (Nivolumab or Opdivo OR ONO-4538 OR ONO 4538 OR MDX-1106 OR MDX 1106 OR MDX1106 OR BMS-936558 OR BMS 936558 OR SCH-900475 OR Nivo):ti,ab

#33 (atezolizumab OR Tecentriq OR RG7446 OR RG-7446 OR MPDL3280A):ti,ab

#34 (avelumab OR MSB0010718C):ti,ab

#35 (durvalumab OR MEDI-4736 OR MEDI4736):ti,ab

#36 #27 or #28 or #29 or #30 or #31 or #32 or #33 or #34 or #35

| **Supplementary Table 2.** Additional characteristics of the included trials | | | |
| --- | --- | --- | --- |
| **Study name** | **Key Inclusion criteria for patient selection** | **Key Exclusion criteria for patient selection** | **Treatment** |
| CheckMate 057,^4^ | Previously treated stage IIIB or IV non-squamous NSCLC; had disease recurrence or progression during or after chemotherapy regimen; aged >18; ECOG PS=0 or 1; had adequate organ function | Autoimmune disease, symptomatic interstitial lung disease; systemic immunosuppression; prior therapy with immune-stimulatory anti-tumour agents; prior docetaxel therapy; untreated, unstable brain metastases | Nivolumab (3mg/kg q2w) vs docetaxel |
|  |  |  |  |
| KEYNOTE-024,^23^ | Untreated stage IV NSCLC without EGFR or ALK mutations; aged >18, ECOG PS=0 or 1; ≥1 measurable lesion according to RECIST, v 1.1; a life expectancy of at least 3 months, and PD-L1 tumor proportion score ≥ 50% . | Receiving systemic glucocorticoids or other immunosuppressive treatment; untreated brain metastases, active autoimmune disease, active interstitial lung disease, or history of pneumonitis requiring systemic steroids. | Pembrolizumab (200mg q3w) vs CTx |
|  |  |  |  |
| JAVELIN Lung 200,^21^ | Previously treated stage IIIB, IVor recurrent NSCLC; disease progression after previous platinum doublet treatment; aged >18; ECOG PS=0 or 1; an estimated life expectancy of ≥3months, and adequate organ function. | EGFR or ALK mutation; previously received a drug targeting a T-cell regulatory protein or systemic anticancer treatment after disease progression with platinum-based combination therapy; untreated brain metastases, persisting toxicity after previous treatment, or other clinically significant diseases. | Avelumab (10mg/kg q2w) vs docetaxel |
|  |  |  |  |
| OAK,^6^ | Previously treated NSCLC, measurable disease per PECIST v1.1, ECOG PS=0 or 1 | Autoimmune disease; had received previous treatment with docetaxel, CD137 agonists, anti-CTLA4, or anti-PD-L1/PD-1 treatment. | Atezolizumab (1200mg q3w) vs docetaxel |
| KEYNOTE-189,^7^ | Untreated metastatic nonsquamous NSCLC without EGFR or ALK mutations; aged >18; ECOG= 0 or 1; ≥1 measurable lesion according to RECIST, v 1.1; available tumor PD-L1 status | symptomatic CNS metastases; a history of noninfectious pneumonitis that required receiving systemic immunosuppressive treatment, active autoimmune disease; previously received ≥ 30 Gy of radiotherapy to the lung in the previous 6 months. | Pembrolizumab (200mg q3w) plus CTx vs CTx |
| CheckMate 227,^22^ | Stage IV or recurrent NSCLC; aged >18; ECOG PS=0 or 1; no previous systemic therapy; | EGFR or ALK mutations, autoimmune disease, or untreated or symptomatic CNS metastases; glucocorticoids <2 weeks before randomization | Nivolumab (3mg/kg q2w) plus ipilimumab vs CTx |
|  |  |  |  |
| CheckMate 078,^24^ | Stage IIIB or IV or recurrent NSCLC progressing during or after one previous platinum-based doublet chemotherapy regimen; aged >18, ECOG PS=0 or 1, measurable lesion according to RECIST, v 1.1; patients with treated, stable brain metastases were eligible. | Patients with EGFR or ALK mutation–positive; active autoimmune disease, symptomatic interstitial lung disease, and systemic immunosuppression; patients previously treated with an antitumor vaccine, immunostimulatory anti-tumor agent, immune checkpoint inhibitor, or docetaxel | Nivolumab (3mg/kg q2w) vs docetaxel |
|  |  |  |  |
| SHR-1210-303,^25^ | Stage IIIB-IV non-squamous NSCLC; no prior systemic treatment; measurable disease per RECIST 1.1 criteria; aged >18; ECOG PS=0 or 1; life expectancy of at least 3 months. | EGFR and/or ALK mutation; active CNS metastases; autoimmune disease; requiring systemic immunosuppressive medications < 14 days before administration; a history of interstitial lung disease, active pulmonary tuberculosis; known HIV-positive, active hepatitis B or C; clinically significant cardiovascular and cerebrovascular diseases | Camrelizumab (200mg q3w) plus CTx vs CTx |
| Checkmate 9LA,^27^ | Stage IV or recurrent NSCLC; no previous systemic therapy; No EGFR or ALK mutations; ECOG PS=0 or 1; aged >18; measurable lesion according to RECIST, v 1.1; available tumor PD-L1 status | untreated CNS metastases; active, known or suspected autoimmune disease; symptomatic interstitial lung disease, receiving systemic immunosuppressive treatment; known HIV-positive, active hepatitis B or C | Nivolumab (360mg q3w) plus ipilimumab plus CTx vs CTx |
| IMpower133,^8^ | extensive-stage SCLC; aged >18; measurable disease per RECIST 1.1 criteria; ECOG PS=0 or 1; no previous systemic therapy; patients with treated, stable brain metastases were eligible. | a history of autoimmune disease; previous treatment with CD137 agonists or immune-checkpoint blockade therapies; treatment with systemic immunosuppressive medications; known HIV-positive, active hepatitis B or C | Atezolizumab (1200 mg q3w)+ CTx vs CTx |
| CASPIAN,^26^ | extensive-stage SCLC; aged >18; measurable disease per RECIST 1.1 criteria; ECOG PS=0 or 1; no previous systemic therapy; an estimated life expectancy of ≥3 months; a bodyweight of more than 30 kg; adequate organ and bone marrow function; and suitability for first-line platinum-based chemotherapy. | a history of chest radiotherapy or active primary immunodeficiency; active or previous autoimmune or inflammatory disorders; paraneoplastic syndrome of autoimmune nature requiring systemic treatment; and uncontrolled, concurrent illness or active infections. patients with treated, stable brain metastases were eligible | Durvalumab (1500 mg q4w) plus CTx +/-tremelimumab vs CTx |
| Keynote 604,^28^ | extensive-stage SCLC; aged >18; measurable disease per RECIST 1.1 criteria; ECOG PS=0 or 1; no previous systemic therapy; an estimated life expectancy of ≥3 months; and adequate organ function. patients with treated, stable brain metastases were eligible | a history of (non-infectious) pneumonitis that required steroids or has current pneumonitis; symptomatic interstitial lung disease, and systemic immunosuppression; known HIV-positive, active hepatitis B or C; previous treatment with CD137 agonists or immune-checkpoint blockade therapies | Pembrolizumab (200 mg q3w) plus CTx vs CTx |
| ORIENT-11,^30^ | Stage IV or recurrent NSCLC; no previous systemic therapy; No EGFR or ALK mutations; ECOG PS=0 or 1; aged >18; measurable lesion according to RECIST, v 1.1; an estimated life expectancy of ≥3 months; adequate organ function | Pulmonary radiation therapy of >30 Gy within 6 months; active autoimmune diseases or infections requiring systemic treatment; active central nervous system (CNS) metastasis and/or cancerous meningitis; known HIV-positive, active hepatitis B or C | Sintilimab (200mg, q3w) plus CTx vs CTx |
| EMPOWER-Lung 1,^29^ | Stage IIIB-IV NSCLC; no prior systemic treatment; measurable disease per RECIST 1.1 criteria; aged >18; ECOG PS=0 or 1; life expectancy of at least 3 months. | EGFR and/or ALK mutation; active or untreated brain metastases; autoimmune disease; requiring systemic immunosuppressive medications < 14 days before administration; a history of interstitial lung disease, active pulmonary tuberculosis; known HIV-positive, active hepatitis B or C; prior therapy with anti-PD 1 or anti-PD L1 | Cemiplimab (350mg q3w) vs CTx |

| **Supplementary Table 3.** Risk of bias by the Cochrane Collaboration Tool | | | | | | | |
| --- | --- | --- | --- | --- | --- | --- | --- |
| **Study name** | **Random sequence generation**  **(selection bias)** | **Allocation concealment**  **(selection bias)** | **Blinding of participants and personnel (performance bias)** | **Blinding of outcome assessors**  **(detection bias)** | **Incomplete outcome data**  **(attrition bias)** | **Selective outcome reporting**  **(reporting bias)** | **Other sources of bias**  **(other bias)** |
| CheckMate 057,^4^ | Low | Low | High | High | Low | Low | Low |
| KEYNOTE-024,^23^ | Low | Low | High | Low | Low | Low | Low |
| JAVELIN Lung 200,^21^ | Low | Low | High | Low | Low | Low | Low |
| OAK,^6^ | Low | Low | High | High | Low | Low | Low |
| KEYNOTE-189,^7^ | Low | Low | Low | Low | Low | Low | Unclear |
| CheckMate 227,^22^ | Low | Low | High | Low | Low | Low | Low |
| CheckMate 078,^24^ | Low | Low | High | High | Low | Low | Low |
| SHR-1210-303,^25^ | Low | Low | High | Low | Low | Low | Low |
| Checkmate 9LA,^27^ | Low | Low | High | Low | Unclear | Unclear | Low |
| IMpower133,^8^ | Low | Low | Low | Low | Low | Low | Low |
| CASPIAN,^26^ | Low | Low | High | High | Low | Low | Low |
| Keynote 604,^28^ | Low | Low | Low | Low | Low | Low | Low |
| ORIENT-11,^30^ | Low | Low | Low | Low | Unclear | Unclear | Low |
| EMPOWER-Lung 1,^29^ | Low | Low | High | Low | Unclear | Unclear | Low |

| **Supplementary Table 4.** Results of Sensitivity Analyses | | | | |
| --- | --- | --- | --- | --- |
| **Removed trials** | **Outcome** | **No. of**  **studies** | **No. of**  **patients** | **HR (95% CI)** |
| Random-effects model | OS | 13 | 8280 | 0.96 [0.78–1.18] |
| Fixed-effects model | OS | 13 | 8280 | 0.95 [0.80, 1.12] |
| Random-effects model | PFS | 10 | 5174 | 0.97 [0.79–1.20] |
| Fixed-effects model | PFS | 10 | 5174 | 0.97 [0.79 –1.20] |
| KEYNOTE-024,^23^ |  |  |  |  |
| Before sensitivity analysis | OS | 13 | 8280 | 0.96 [0.78–1.18] |
| After sensitivity analysis | OS | 12 | 7975 | 0.96 [0.78–1.19] |
| Before sensitivity analysis | PFS | 10 | 5174 | 0.97 [0.79–1.20] |
| After sensitivity analysis | PFS | 9 | 4869 | 0.97 [0.78–1.20] |
| CheckMate 227,^22^ |  |  |  |  |
| Before sensitivity analysis | OS | 13 | 8280 | 0.96 [0.78–1.18] |
| After sensitivity analysis | OS | 12 | 7114 | 0.98 [0.78–1.24] |
| CASPIAN,^26^ |  |  |  |  |
| Before sensitivity analysis | OS | 13 | 8280 | 0.96 [0.78–1.18] |
| After sensitivity analysis | OS | 12 | 7743 | 0.95 [0.76–1.19] |

## Supplementary Figures

**Supplementary Figure 1.** Funnel plot comparing hazard ratios for overall survival and progression-free survival

**A**

**B**

Funnel plots depicting potential presence of publication bias for the outcome overall survival (A) and progression-free survival (B). Each study’s effect estimate plotted against its standard error. The outer solid lines represent the confidence interval boundary within which 95% of studies are expected to lie in the absence of bias or heterogeneity. The solid vertical line represents the summary treatment effect, derived using random-effects meta-analysis.

**Supplementary Figure 2.** The pooled ratio of OS-HRs in BMs versus non-BMs patients


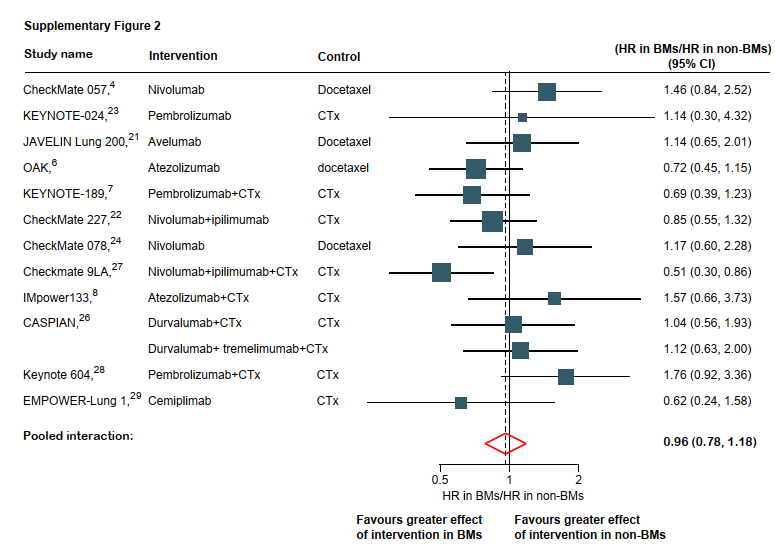


Description: Squares indicate the study-specific interaction HR, that is the ratio of the reported HRs in BMs patients and in non-BMs patients. Size of the square is proportional to the precision of the estimate (i.e. the inverse of the variance). Horizontal lines indicate the 95% CI. Diamonds indicate the meta-analytic pooled HRs with their corresponding 95% CIs. The dashed vertical line indicates the pooled interaction HR, and the solid vertical line indicates an interaction HR of 1, which is the null-hypothesis value (i.e. no difference between BMs and non-BMs patients of the immunotherapy effect).

Abbreviations: OS: overall survival; BMs: brain Metastases; HR, Hazard ratio; CI, confidence interval.

**Supplementary Figure 3.** The pooled ratio of PFS-HRs in BMs versus non-BMs patients


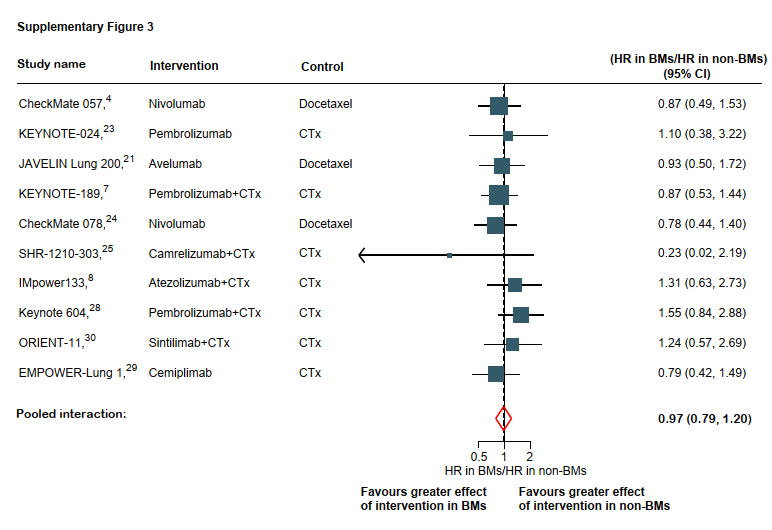


Description: Squares indicate the study-specific interaction HR, that is the ratio of the reported HRs in BMs patients and in non-BMs patients. Size of the square is proportional to the precision of the estimate (i.e. the inverse of the variance). Horizontal lines indicate the 95% CI. Diamonds indicate the meta-analytic pooled HRs with their corresponding 95% CIs. The dashed vertical line indicates the pooled interaction HR, and the solid vertical line indicates an interaction HR of 1, which is the null-hypothesis value (i.e. no difference between BMs and non-BMs patients of the immunotherapy effect).

Abbreviations: PFS: progression-free survival; BMs: brain Metastases; HR, Hazard ratio; CI, confidence interval.
